# SA and NHP glucosyltransferase *UGT76B1* affects plant defense in both *SID2*- and *NPR1*-dependent and independent manner

**DOI:** 10.1007/s00299-024-03228-5

**Published:** 2024-05-23

**Authors:** Wei Zhang, Rafał Maksym, Elisabeth Georgii, Birgit Geist, Anton R. Schäffner

**Affiliations:** 1 Institute of Biochemical Plant Pathology, Department of Environmental Sciences, Helmholtz Zentrum München, Neuherberg, Germany; 2 College of Life Sciences, Jiangsu University, Jiangsu, People’s Republic of China

**Keywords:** Plant pathogen defense, Salicylic acid, N-hydroxypipecolic acid, *SID2*, *NPR1*, *FMO1*

## Abstract

**Key message:**

The small-molecule glucosyltransferase loss-of-function mutant *ugt76b1* exhibits both *SID2*- or *NPR1*-dependent and independent facets of enhanced plant immunity, whereupon *FMO1* is required for the *SID2* and *NPR1* independence.

**Abstract:**

The small-molecule glucosyltransferase UGT76B1 inactivates salicylic acid (SA), isoleucic acid (ILA), and N-hydroxypipecolic acid (NHP). *ugt76b1* loss-of-function plants manifest an enhanced defense status. Thus, we were interested how *UGT76B1* genetically integrates in defense pathways and whether all impacts depend on SA and NHP. We study the integration of *UGT76B1* by transcriptome analyses of *ugt76b1*. The comparison of transcripts altered by the loss of *UGT76B1* with public transcriptome data reveals both SA-responsive, *ISOCHORISMATE SYNTHASE 1/SALICYLIC ACID INDUCTION DEFICIENT 2* (*ICS1*/*SID2*)- and *NON EXPRESSOR OF PR GENES 1* (*NPR1*)-dependent, consistent with the role of UGT76B1 in glucosylating SA, and SA-non-responsive, *SID2*/*NPR1*-independent genes. We also discovered that *UGT76B1* impacts on a group of genes showing non-SA-responsiveness and regulation by infections independent from *SID2*/*NPR1*. Enhanced resistance of *ugt76b1* against *Pseudomonas syringae* is partially independent from *SID2* and *NPR1*. In contrast, the *ugt76b1*-activated resistance is completely dependent on *FMO1* encoding the NHP-synthesizing *FLAVIN-DEPENDENT MONOOXYGENASE 1*). Moreover, *FMO1* ranks top among the *ugt76b1*-induced *SID2*- and *NPR1*-independent pathogen responsive genes, suggesting that *FMO1* determines the *SID2*- and *NPR1*-independent effect of *ugt76b1*. Furthermore, the genetic study revealed that *FMO1*, *ENHANCED DISEASE SUSCEPTIBILITY 1* (*EDS1*), *SID2*, and *NPR1* are required for the SA–JA crosstalk and senescence development of *ugt76b1*, indicating that *EDS1* and *FMO1* have a similar effect like stress-induced SA biosynthesis (*SID2*) or the key SA signaling regulator *NPR1*. Thus, *UGT76B1* influences both *SID2*/*NPR1*-dependent and independent plant immunity, and the *SID2*/*NPR1* independence is relying on *FMO1* and its product NHP, another substrate of UGT76B1.

**Supplementary Information:**

The online version contains supplementary material available at 10.1007/s00299-024-03228-5.

## Introduction

Salicylic acid (SA) and N-hydroxypipecolic (NHP) acid play a central and concerted role in establishing *Arabidopsis* pathogen defense mainly against (hemi)-biotrophic pathogens such as *Pseudomonas syringae*. They coordinately affect both local immunity and systemic acquired resistance (SAR). Thereby, NHP is indispensable for triggering of SAR, whereas SA is required for a fully established local and systemic defense (Ding and Ding 2020; Vlot et al. 2009). Their biosynthesis is also highly interconnected. ICS1/SID2 is responsible for synthesizing SA in *Arabidopsis thaliana* (Dewdney et al. 2000; Nawrath and Metraux 1999). FMO1 converts pipecolic acid into NHP and orchestrates defense via both *SID2*- (SA-)-dependent and independent pathways during SAR (Bernsdorff et al. 2016; Hartmann and Zeier 2018; Hartmann et al. 2018; Zeier 2021). Several players organizing the interplay of SA and NHP biosynthesis and signaling have been identified. A cascade of both positive and negative transcription factors channels immune perception to enhance transcription of biosynthetic genes and the immune regulators *EDS1* and *PHYTOALEXIN-DEFICIENT 4* (*PAD4*), which are required for both SA and NHP formation (Bartsch et al. 2006; Huang et al. 2020; Zeier 2021; Shields et al. 2022). NPR1 is a shared, key downstream regulator of SA- and NHP-mediated local and systemic responses (Ding and Ding 2020; Ding et al. 2020; Vlot et al. 2009; Zheng and Dong 2013). While SA and NHP act mostly synergistic, the SA pathway usually exerts an antagonistic effect on the JA pathway. This antagonism requires *NPR1* (Vlot et al. 2009). However, *NPR1*-independent and also *SID2*- (SA-) independent regulation of pathogen defense plays a vital function in regulating defense as well. An *NPR1*-independent defense response was found in several mutants, such as *ssi1*, *ssi2*, *cpr5*, *cpr6*, *acd6*, and *cdd1* (Bowling et al. 1997; Clarke et al. 1998; Rate et al. 1999; Shah et al. 1999, 2001; Swain et al. 2011, 2015). The lesion-mimic *Arabidopsis* mutant *syp121 syp122* suggested that some SA-independent signals are mediated by *FMO1* (Zhang et al. 2008). Furthermore, the activity and mutual enhancement of SA and NHP is also regulated at the metabolic level. Four independent studies suggested that the small-molecule glucosyltransferase *UGT76B1* can conjugate and inactivate SA and NHP in a competitive manner, in fact in concert with another immune-stimulating compound, isoleucic acid (ILA) (Bauer et al. 2021; Cai et al. 2021; Holmes et al. 2021; Mohnike et al. 2021). Thereby, *UGT76B1* plays a decisive role in the interplay of SA, NHP, and ILA balancing a low-level defense status in naïve, non-infected plants, whereas it attenuates defense upon infection (Bauer et al. 2021; Holmes et al. 2021; Mohnike et al. 2021). Consequently, *UGT76B1* had been shown to suppress defense against *Pseudomonas syringae* accompanied by downregulation of SA marker genes such as *PR1* and upregulation of the mostly antagonistic JA pathway markers such as *VSP2* and to delay senescence (von Saint Paul et al. 2011). To integrate the action of *UGT76B1* into these defense pathways, we compared a non-targeted gene expression analysis of *ugt76b1* with public expression data revealing both SA- and non-SA-responsive genes. Further comparison with public data revealed many *SID2*- and also *NPR1*-independently regulated genes among non-SA responsive group of *ugt76b*1, with *FMO1* ranking top. Consistently, the resistance of *ugt76b1* against *Pseudomonas syringae* is partially independent from *SID2* and *NPR1*, whereas the resistance against *Pseudomonas* is known to completely rely on *FMO1* (Bauer et al. 2021). Further genetic analyses showed that the induction of the SA marker *PR1*, the suppression of the JA marker *VSP2* by *ugt76b1*, and the senescence phenotype of *ugt76b1* are mainly dependent on *SID2*, *NPR1*, *EDS1*, and *FMO1*. Thus, the impact of *ugt76b1* may be mediated through the upregulation of SA (*SID2*) and NHP (*FMO1*) pathways. The *SID2*- and *NPR1*-dependent mode is consistent with the role of *UGT76B1* in glucosylating SA, while the *SID2*- and *NPR1*-independent regulation is mediated through FMO1 and its product NHP in accordance with the dual action of UGT76B1 to glucosylate and inactivate SA and NHP.

## Materials and methods

### Plant material and growth condition

*Arabidopsis thaliana* plants (Col-0 accession) were grown in soil under a regime of 14 h light (45–60 µmol m^−2^ s^−1^) and 20 °C; temperature was reduced to 18 °C in the dark phase with 75% relative humidity. Mutant *Arabidopsis* lines were obtained from the *Arabidopsis* stock center (*fmo1*, SALK_026163; *ora59*, GK_061A12; *ugt76b1*, SAIL 1171A11) (Bartsch et al. 2006; Scholl et al. 2000; von Saint Paul et al. 2011) and from colleagues (*eds1-2*, Corina Vlot-Schuster, Bayreuth; *jin1*, Susanne Becker, Würzburg; *npr1-1*, Corina Vlot-Schuster, Bayreuth; *sid2-1*, Christiane Nawrath, Lausanne) (Bartsch et al. 2006; Berger et al. 1996; Cao et al. 1994; Nawrath and Metraux 1999). All double mutants were generated by genetic crossing and then selected by PCR-based genotyping or CAPS polymorphisms (Cao et al. 1994; Nawrath and Metraux 1999; Rosso et al. 2003; Sessions et al. 2002).

### *Pseudomonas* infection

The biotrophic pathogen *Pseudomonas syringae* pv *tomato* DC3000 (*Ps-vir*) was used in this project. Bacteria were streaked out onto fresh solid King’s B medium containing 50 μg mL^−1^ kanamycin and grown for 2 days at 28 °C. A single colony was picked and grown overnight in liquid King’s B medium with antibiotic at 28 °C at a shaker speed of 170 rpm. When bacteria reached late log phase of growth (OD_600_ = 0.6–1.0), they were diluted to 5 × 10^5^ cfu mL^−1^ in 10 mM MgCl_2_ for the inoculation of plants. An OD_600_ = 0.001 corresponds to 5 × 10^5^ colony-forming units mL^−1^. Four leaves of 5- to 6-week-old *Arabidopsis* (6^th^–11th leaves) were labeled by a marker pen and infiltrated with the diluted bacteria using a 1 mL syringe. Control plants were infiltrated with 10 mM MgCl_2_ as mock treatment. Bacteria (cfu cm^−2^) were quantified 0 and 3 days after inoculation. To determine the bacteria number after inoculation, leaf discs with an area of 0.20 cm^2^ were cut using the lid of a 0.5 mL Eppendorf tube. Two leaf discs from each individually infected plant were harvested. Six leaf discs from three individual plants were pooled as one biological replicate. In total, at least four independent biological replicates were analyzed. Bacterial numbers were calculated according to Katagiri et al. (2002).

### Real-time PCR

Plants were grown on soil employing 16 h light/8 h darkness regime. Total RNA was extracted from about 60 mg of rosette leaf powder using RNeasy Plant Mini kit (Qiagen, Germany) and dissolved in 30 µL of RNase/DNase free water. Quality and concentration were analyzed using the Nanodrop ND-1000 spectrophotometer (Kisker-Biotech, Germany). Primers for RT-qPCR were designed using the Primer Express 3.0 software (Applied Biosystems, Germany) according to the reference mRNA sequences (Supplementary Table 6). The first-strand cDNA was transcribed from 1 µg total RNA using QuantiTect Reverse Transcription Kit (Qiagen, Germany). The Applied Biosystems (Germany) 7500 real-time PCR system was used for quantitative PCR recording SYBR Green fluorescence (Thermo Scientific or Bioline, Germany). Each sample was repeated with two technical replicates. *UBQ5* (At3g62250) and *S16* (At5g18380, At2g09990) were chosen as two reference genes to normalize the relative abundance of the genes of interest according to GeNorm analysis (Vandesompele et al. 2002). Arithmetic means and standard errors from log_10_-transformed data of RT-qPCR data from more than three independent experiments were statistically assessed by an “R” software package employing two-way analysis of variance (ANOVA; linear mixed effect models) followed by post hoc Tukey’s HSD test correction.

### Untargeted microarray analysis and data analysis

*Arabidopsis* plants were grown under a 14 h light/10 h dark regime at 45–60 µmol m^−2^ s^−1^ fluorescent light. The transcriptome analysis of *ugt76b1-1*, *UGT76B1-OE-7* and wild type (accession Columbia) was performed using *A. thaliana* Agilent At8×60 K one-color microarrays (Design ID: 29132, A-GEOD-16892) (Agilent, Germany) according to the manufacturer’s instructions. The assays were done as previously described (Georgii et al. 2017). Three biological replicates of each genotype were analyzed. Leaves from eight 4-week-old *Arabidopsis* plants were harvested to be pooled as one replicate. The “One-color Microarray-Based Gene Expression Analysis-Low Input Quick Amp Labeling” according to Agilent G4140-90040 was employed. The fluorescent signals from the arrays were analyzed by the Agilent Feature Extraction Software (Agilent, Germany). Probes were mapped to AGI loci using TAIR10 (Berardini et al. 2015). The R software package Limma was used to perform quantile normalization and compute differential gene expression. Transcripts with more than twofold changes compared to the control (Col) and a significant change based on corrected p values smaller than 0.05 were chosen for further analysis. BioMaps (www.virtualplant.org) version 1.3 was used for functional analysis of gene lists. Over-representation of Gene Ontology terms (https://www.arabidopsis.org/tools/go_term_enrichment.jsp) was assessed using binomial-test p values. A corrected p value (with Bonferroni correction) smaller than 0.016 was considered to indicate a significant over-representation. Genevestigator (https://www.genevestigator.com/gv/) was used to compare the expression pattern of genes of interest with public data.

## Results

### *UGT76B1* expression negatively regulates defense-responsive genes

**Fig. 1:**
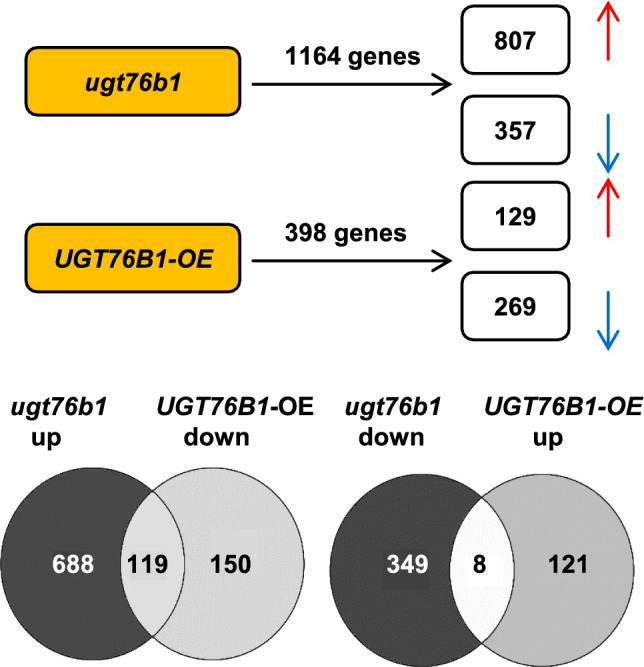
Transcriptional reprogramming of genes by *UGT76B1* expression. Microarray analysis was performed using *A. thaliana* Agilent At8×60 K one-color microarrays. Gene expression was compared among *ugt7b1-1*, *UGT76B1-OE-7,* and wild type (accession Columbia). Differentially expressed genes by loss-of-function of UGT76B1 and constitutive overexpression of *UGT76B1* are indicated. Genes induced or suppressed more than twofold are indicated as *red* and *blue arrow*, respectively. The Venn diagrams display the overlaps between genes oppositely regulated by *ugt76b1* and *UGT76B1-OE* (colour figure online)

**Fig. 2:**
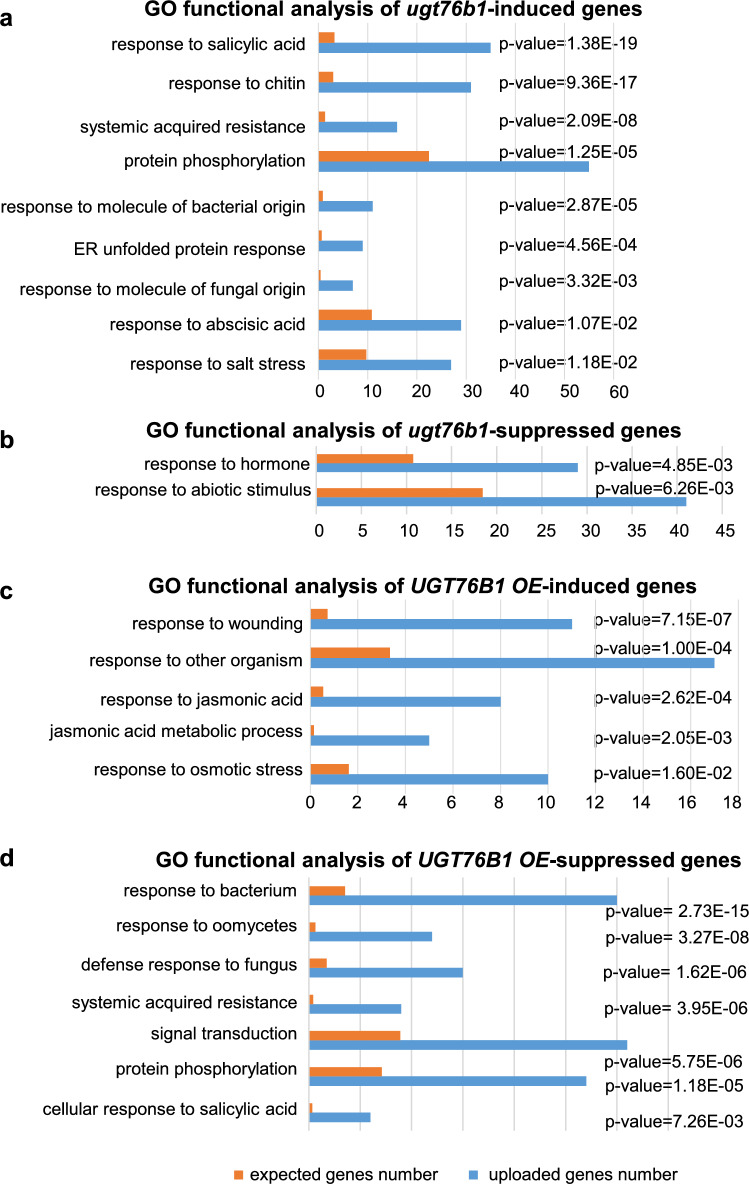
GO term-based functional analysis of genes deregulated by the altered expression of UGT76B1. **a** Eight hundred and seven genes induced more than twofold and **b** three hundred and fifty-seven genes suppressed by loss-of-function of *UGT76B1* were grouped according to GO term analysis. **c** One hundred and twenty-nine genes induced more than twofold, and **d** two hundred and sixty-nine genes suppressed more than twofold by UGT76B1 overexpression were grouped according to GO term analysis. GO biological process assignments by Panther at TAIR (https://www.arabidopsis.org/tools/go_term_enrichment.jsp) were used to calculate the *P* value of over-representation by a binomial test (with Bonferroni correction) with a cutoff of 0.016 for **a** and **c** and a cutoff of 0.010 for **b** and **d**

*UGT76B1* has been shown to glucosylate and inactivate the three immune-modulatory ILA, SA, and NHP (Bauer et al. 2021; Holmes et al. 2021; Mohnike et al. 2021). To identify genes and pathways that are specifically affected by the action of *UGT76B1*, we compared differential gene expression pattern of the loss-of-function mutant *ugt76b1-1* and a constitutively *UGT76B1-*overexpressing line (*UGT76B1-OE*) relative to the wild-type Columbia (Col) by a non-targeted analysis employing the Agilent G4140-90040 *A. thaliana* microarray based on the TAIR10 annotation. The expression of 1164 genes was altered at least twofold (adjusted *P* ≤ 0.05) by *ugt76b1-1* compared to Col. Among these genes, 807 genes were upregulated and 357 genes were downregulated (Fig. 1). The constitutive overexpression of *UGT76B1* (*UGT76B1-OE*) led to a change in the expressions of 398 genes in comparison to wild type (at least twofold change, *P* ≤ 0.05). Among these 398 genes, 129 genes were induced, whereas 269 genes were suppressed (Fig. 1). According to TAIR gene ontology (GO) function analysis, genes induced by *ugt76b1-1* are enriched in the group of “response to salicylic acid”, “response to chitin”, “systemic acquired resistance”, “protein phosphorylation”, “response to molecule of bacterial origin”, “ER unfolded protein response”, “response to molecule of fungal origin”, “response to abscisic acid”, and “response to salt stress” sorted by adjusted P value from smallest to largest (*P* ≤ 0.012) (Fig. 2a). However, only the groups of “response to hormone” and “response to abiotic stimulus” have been shown to be enriched by *ugt76b1*-suppressed genes (*P* ≤ 0.01) (Fig. 2b). In addition, the groups of “response to wounding”, “response to other organism”, “response to jasmonic acid”, “jasmonic acid metabolic process”, and “response to osmotic stress” have been shown to be over-represented by *UGT76B1* overexpression-induced genes sorted by adjusted P value from smallest to largest (*P* ≤ 0.01) (Fig. 2c). Moreover, genes suppressed by overexpression of *UGT76B1* are related to “response to bacterium”, “response to oomycetes”, “defense response to fungus”, “systemic acquired resistance”, “signal transduction”, “protein phosphorylation”, and “cellular response to salicylic acid stimulus” sorted by P value from smallest to largest (*P* ≤ 0.01) (Fig. 2d). A Venn diagram indicates that 127 genes were oppositely regulated by the loss-of-function *vs*. the ectopic expression of *UGT76B1*. The vast majority, 119 genes, were upregulated by the *ugt76b1* knockout and downregulated by the *UGT76B1* overexpression (Fig. 1; Supplementary Table 7). Only eight genes were induced by overexpression of *UGT76B1* but suppressed by the loss-of-function of *UGT76B1* (Fig. 1; Supplementary Table 8). The common 119 *ugt76b1* up- and *UGT76B1*-*OE* downregulated genes are enriched in the groups of “response to salicylic acid”, “defense response to bacterium”, “systemic acquired resistance”, “defense response to fungus”, “response to molecule of bacterial origin”, “response to oomycetes”, “signal transduction”, “cellular response to oxygen-containing compound”, “protein phosphorylation”, “response to lipid”, and “response to inorganic substance” sorted by P value from smallest to largest (*P* ≤ 0.01) (Supplementary Fig. 1). Among the common eight genes suppressed by *ugt76b1*, however, induced by *UGT76B1*-*OE*, At4g23600 encoding a tyrosine transaminase family protein is responsible for regulating the JA pathway (Lopukhina et al. 2001) (Supplementary Table 8), consistent with the suppression of the JA pathway by *ugt76b1* and its upregulation by *UGT76B1-OE*. This strongly suggests that UGT76B1’s function mainly leads to suppression of a set of defense-responsive genes.

### SA-responsive and non-responsive genes of *ugt76b1* show both *SID2*- and *NPR1*-dependent and -independent regulation

UGT76B1 attenuates basal and induced defense responses, glucosylating SA, NHP, and ILA. NHP and possibly its biosynthetic precursor pipecolic acid (Pip) amplify the biosynthesis of SA and regulate both *SID2*-dependent and independent responses (Bernsdorff et al. 2016; Hartmann and Zeier 2018; Hartmann et al. 2018; Bauer et al. 2021). ILA application can increase Pip and NHP abundance (Bauer et al. 2021) suggesting the existence of both *SID2*-dependent and independent regulations, as well. We therefore hypothesize that genes induced by *ugt76b1* may be classified as SA-responsive and non-responsive. Accordingly, differential gene expression of *ugt76b1* was compared with public expression data involving SA responses. The genes induced by *ugt76b1* were compared to Affymetrix microarray-based public data deposited at Genevestigator (https://www.genevestigator.com/gv/plant.jsp; Zimmermann et al. 2005). These experiments comprise the response to exogenous SA or benzothiadiazol (BTH, a functional analogue of SA) treatment, to infection by different strains of *Pseudomonas syringae* (*P. syringae* pv. *tomato* DC3000, *P. syringae* pv. *tomato* DC3000 *avrRptm1*, *P. syringae* pv. *phaseolicola*, and *P. syringae* pv. *maculicola*) of wild type (*vs*. mock), to infection of *sid2* by *P. syringae* pv. *tomato* avirulent strain DC3000 *avrB*, and to *P. syringae* pv. *maculicola* infection of *npr1-1* or *sid2*. One thousand and six Affymetrix features matched the 1164 genes identified in our study. They were first classified into three groups according to the strength in response to SA and BTH treatment. A group of 494 genes was induced or suppressed by SA and BTH more than twofold (384 induced and 110 suppressed transcripts; log_2_FC ≥ 1.0), and 152 genes showed an intermediate change of 1.5-to-2-fold (117 induced and 45 suppressed transcripts; 0.58 ≤ log_2_FC < 1.0) indicating a potential regulation by SA or BTH, whereas 350 genes were altered by a factor of less than 1.5 (219 induced and 131 suppressed features; log_2_FC < 0.58). This latter group was, therefore, classified as non-SA responsive. The genes were further sorted according to induction to pathogen infections. The majority of the SA-responsive genes are related to pathogen defense. Among the SA-responsive group, the 368 defense-responsive genes account for 93% of 384 *ugt76b1*-induced genes, and 92 defense-responsive genes overlap with more than 80% of 110 *ugt76b1*-suppressed genes (Table 1). Among the potentially SA-responsive group, 84% of *ugt76b1*-induced and 58% of *ugt76b1*-suppressed genes were categorized as defense-related genes (Table 1). A much lower frequency of defense-related transcripts was observed among the non-SA responsive genes. However, still more than half, i.e., 57% of the 219 *ugt76b1*-induced, non-SA-responsive genes, were associated with pathogen defense, whereas only 20% of the 131 *ugt76b1*-suppressed genes classified to this category (Table 1). The altered transcription in response to various *Pseudomonas* infection experiments, yet lacking response to SA or BTH treatment further supports the association with defense in a non-SA responsive group (Table 1). Thus, *UGT76B1* suppresses a set of defense-responsive genes even among the non-SA responsive group.

**Table 1:** Genes uploaded into Genevestigator were classified into non-SA responsive, SA-responsive, and partially SA-responsive groups

Category	Gene numbers	Defense-related genes value and percentage
Non-SA responsive (<1.5 fold SA induction)	219	125 (57%)
Non-SA responsive (<1.5 fold SA suppression)	131	26 (20%)
SA responsive (≥2 folds SA induction)	384	368 (93%)
SA responsive (≥2 folds SA suppression)	110	92 (84%)
Partial SA responsive (≥1.5 but <2 folds SA induction)	117	98 (84%)
Partial SA responsive (≥1.5 but <2 folds SA suppression)	45	26 (58%)

One thousand and six genes out of 1164 *ugt76b1*-altered genes matched with Affymetrix probes in Genevestigator. BTH is a functional analogue of SA and thus considered similar to SA treatment. Genes were classified into six groups according to the strength responding to SA or BTH treatment. Furthermore, genes responding to any pathogen stimulus were taken as defense-related genes, as shown in Supplementary Tables 1, 2 and 3

SA responses are critically dependent on the biosynthetic function of *SID2* and the signaling node of *NPR1* (Ding and Ding 2020; Vlot et al. 2009). *SID2*-dependent and independent regulations of defense responses had been revealed for the function of Pip and *FMO1* (Bernsdorff et al. 2016; Hartmann and Zeier 2018; Hartmann et al. 2018). To explore the dependence of the *ugt76b1* mutants on *SID2* or *NPR1* in regulating defense, *SID2* and *NPR1* dependence was classified among both SA-responsive and non-SA responsive based on transcriptional responses of *Pseudomonas syringae* pv. *maculicola* infected *npr1-1* or *sid2* (vs. infected Col) or of *P. syringae* pv*. tomato* avirulent strain DC3000 *avrB* infection of *sid2* (*vs.* infected Col or non-infected *sid2*) or the non-infected *sid2* mutants *vs.* non-infected Col (https://www.genevestigator.com/gv/plant.jsp: date; Zimmermann et al. 2005). Most of the SA-responsive genes showed *SID2* and *NPR1* dependence when infected with *Pseudomonas syringae* pv*. maculicola*, especially in the SA-inducible group (Supplementary Table 1). Thus, *UGT76B1* has a major role in suppressing a set of SA-responsive genes regulated via* SID2* and *NPR1*.

Among the non-SA responsive groups of *ugt76b1*-induced genes, most genes showed independence from *SID2* or *NPR1* (Supplementary Table 2). Seventy-one genes showing SID2- and NPR1-independent pathogen responses after comparison with public data were extracted (Table 2; Supplementary Table 2). Indeed, many studies confirm that *FMO1*, *WRKY55*, *KTI1*, *CRK20*, *SRG1*, *CYP71A12*, *RABA4C*, *PUB23*, *MYB15*, *PICBP*, *TPS4*, and *MLO6* are involved in the defense response (Table 2) (Attaran et al. 2008; Chezem et al. 2017; Cui et al. 2021; Ederli et al. 2011; Ellinger et al. 2014; Gruner et al. 2018; Lemarie et al. 2015; Li et al. 2008; Mishina and Zeier 2006; Reddy et al. 2003; Stegmann et al. 2012; Wang et al. 2020). The ethylene signaling responsive proteins *ERF1* and *ERF13* were also induced by *ugt76b1* (Table 2) (Onate-Sanchez and Singh 2002; Solano et al. 1998). *CSAP* is ABA-responsive and positively regulates dark induced senescence (Table 2) (So et al. 2020). *JUL1* participates in the ABA-mediated microtubule disorganization, stomatal closure, and tolerance to drought stress (Yu et al. 2020). This strongly suggests that several defense-related genes altered by *ugt76b1* are linked to aspects other than the *SID2*/*NPR1*-regulated SA pathway.

**Table 2:** Seventy-one defense genes altered by *ugt76b1* showed SID2 and NPR1 independence

AGI code	TAIR	*logFC*	*AveExpr control*	*adj.P.Val*
AT2G44240	DUF239, unknown function	5.82	3.75	1.96E-03
AT1G19250	**FMO1**	4.41	4.16	3.41E-05
AT5G39520	**CSAP**	3.70	4.79	1.24E-04
AT4G13890	EDA36	3.69	4.51	2.88E-04
AT2G40740	**WRKY55**	3.16	5.04	1.67E-06
AT3G10320	MUCI21	2.86	4.02	2.94E-04
AT1G73260	**KTI1**	2.65	4.46	4.87E-03
AT3G24982	RLP40**,** unknown function	2.62	4.75	1.12E-04
AT1G34180	ANAC016	2.58	4.78	2.36E-06
AT3G26470	RPW8 domain protein	2.56	5.31	8.41E-06
AT1G79680	WAKL10	2.52	4.15	3.89E-05
AT1G67980	CCOAMT	2.38	5.38	1.63E-04
AT1G14080	FUT6	2.37	3.61	3.50E-04
AT2G37080	RIP3	2.30	8.10	2.20E-04
AT1G72540	PBL33	2.24	4.34	1.44E-04
AT1G68765	IDA	1.98	3.03	1.81E-03
AT3G13080	MRP3	1.96	7.06	9.43E-04
AT1G67000	ABCC3	1.92	5.71	2.78E-06
AT1G68620	alpha/beta-hydrolases superfamily protein	1.84	6.31	1.60E-03
AT5G23020	IMS2	1.83	7.83	1.21E-04
AT1G30850	RSH4	1.77	3.31	4.10E-02
AT3G56500	serine-rich protein-related	1.77	4.27	9.26E-04
AT4G23280	**CRK20**	1.73	4.29	1.44E-03
AT3G16410	NSP4	1.66	3.27	3.12E-01
AT1G30220	INT2	1.66	4.37	6.89E-04
AT1G17020	**SRG1**	1.61	4.58	4.12E-03
AT1G10070	BCAT-2	1.58	6.84	2.52E-02
AT3G45130	LAS1	1.51	4.22	7.09E-04
AT4G08770	PRX37	1.47	5.38	9.62E-03
AT5G07100	WRKY26	1.46	7.88	4.05E-03
AT4G10120	SPS4F	1.45	10.33	9.50E-03
AT3G23240	**ERF1**	1.43	5.75	1.54E-02
AT4G36430	Peroxidase superfamily protein	1.40	4.51	8.79E-03
AT3G13090	ABCC6	1.40	5.93	2.62E-05
AT2G30750	**CYP71A12**	1.40	4.56	3.26E-03
AT5G47960	**RABA4C**	1.39	4.76	2.03E-02
AT2G36970	UDP-glycosyltransferase UGT86A1	1.38	7.90	1.01E-03
AT4G15610	UPF0497	1.34	6.04	2.95E-03
AT1G26390	BBE4	1.34	4.27	3.88E-02
AT4G23030	MATE efflux family	1.29	6.46	1.67E-03
AT1G69930	GSTU11	1.29	4.11	2.88E-02
AT4G10930	unknown protein	1.27	6.60	2.15E-03
AT5G13330	Rap2.6L	1.25	3.99	5.51E-02
AT2G35930	**PUB23**	1.24	7.54	2.33E-01
AT1G80160	Lactoylglutathione lyase/glyoxalase I family	1.22	4.66	2.23E-02
AT5G42750	BKI1	1.21	6.38	1.34E-01
AT1G68790	LINC3	1.21	9.37	3.75E-03
AT4G26190	HAD superfamily protein	1.17	8.32	5.72E-03
AT5G39720	AIG2L	1.16	4.17	2.20E-04
AT5G16680	PAIPP2	1.15	9.45	6.83E-03
AT3G22460	OASA22	1.14	8.77	6.36E-03
AT5G04020	**PICBP**	1.14	8.17	2.64E-03
AT1G68690	PERK9	1.14	6.04	1.19E-03
AT5G55040	BRD13	1.12	7.14	7.11E-03
AT1G12940	NRT2.5	1.11	3.85	1.98E-02
AT4G14640	CAM8	1.10	3.65	3.30E-03
AT5G25230	Ribosomal protein S5	1.10	6.17	1.48E-01
AT1G61120	**TPS4**	1.08	4.79	2.16E-01
AT1G37130	NR2	1.06	9.49	3.00E-02
AT5G62480	GSTU9	1.06	6.17	2.35E-02
AT3G23250	**MYB15**	1.06	6.23	1.11 E-01
AT5G67310	CYP81G1	1.05	3.97	4.41E-02
AT2G44840	**ERF13**	1.04	7.42	3.25E-01
AT5G10650	**JUL1**	1.04	8.49	1.44E-03
AT1G63750	Disease resistance protein (TIR-NBS-LRR class)	1.04	8.82	2.81E-03
AT1G61560	**MLO6**	1.03	5.91	6.04E-03
AT2G27310	F-box family protein	1.20	8.29	1.17 E-02
AT1G71880	SUC1	1.03	9.38	1.87E-03
AT3G11080	RLP35	1.03	4.31	1.87E-01
AT1G06620	2-oxoglutarate and Fe(II)-dep. oxygenase superfamily protein	1.01	4.97	3.23E-02
AT4G08780	Peroxidase superfamily	1.00	2.93	9.35E-03

Seventy-one non-SA-responsive genes, however still responding to pathogen infections, were classified as SID2- and NPR1-independent according to their missing responsiveness with less than twofold changes found for *sid2* or *npr1* compared to wild type before or after infections and for *sid2* before and after pathogen stimulus (Supplementary Table 2). Genes highlighted in bold were reported to have known functions in categories, such as defense responses, ethylene signaling, or ABA-regulated responses

### The enhanced resistance of *ugt76b1* against *Pseudomonas syringae* DC3000 is partially mediated through *NPR1* and *SID2*

**Fig. 3:**
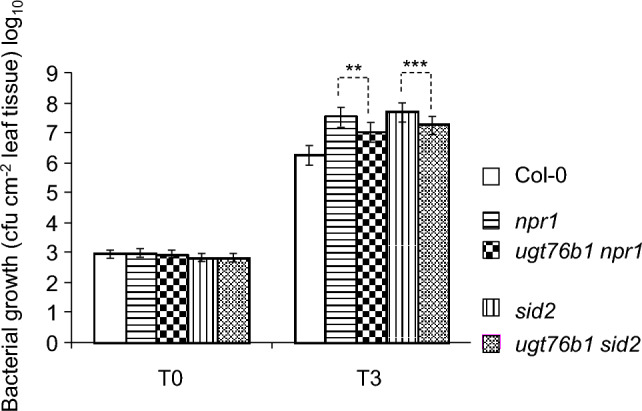
The impact of *UGT76B1* on susceptibility towards *Pseudomonas syringae* DC3000 infection has an *NPR1*- and *SID2*-dependent component. Bacterial growth in inoculated *Arabidopsis* leaves of 4-week-old plants was quantified. Arithmetic means and standard errors from log_10_-transformed data of at least four independent replicates from five separate experiments are displayed. A linear mixed effect model was used to account for random effects from the experiment. For each time point, Tukey post hoc tests were performed to compare all pairs of groups (only specific comparisons of single and matched double mutants are shown). Computations were done in R using the packages nlme and multcomp; ***P value ≤0.001; **P value ≤0.01. No significances were observed among T0

The *ugt76b1* loss-of-function mutant showed activated defense against *Pseudomonas syringae* pv *tomato* DC3000 (von Saint Paul et al. 2011), which was attributed to the glucosylation and inactivation of the immune-stimulatory ILA, SA, and NHP by UGT76B1 (Bauer et al. 2021; Holmes et al. 2021; Mohnike et al. 2021). Pip mediates both *SID2*-dependent and independent defenses via* FMO1* encoding the NHP-synthesizing enzyme (Bernsdorff et al. 2016; Vlot et al. 2021). Finally, *NPR1*, downstream of stress-induced SA biosynthesis, is the master regulator of the SA defense pathway. To exam the roles of *SID2* and *NPR1* in *ugt76b1-*activated immunity, we compared *P. syringae* infection of *ugt76b1-1 npr1* and *ugt76b1-1 sid2* double mutants with wild type and the corresponding *npr1* and *sid2* single mutants. Both *ugt76b1 npr1* and *ugt76b1 sid2* showed enhanced bacterial growth compared to Col plants, indicating that the higher resistance of *ugt76b1* is positively regulated by and dependent on both *NPR1* and *SID2* (Fig. 3). However, when compared to the *npr1* single mutant, *ugt76b1 npr1* showed reduced bacterial proliferation, suggesting a partially *NPR1*-independent enhancement of resistance due to the loss of *UGT76B1*. When compared to the *sid2* mutant, further resistance gained by *ugt76b1 sid2* indicated a partial *SID2*-independent regulation as well (Fig. 3).

### The antagonistic impact of *ugt76b1* on the SA and JA pathways depends on *EDS1*, *NPR1*, and *FMO1*

**Fig. 4:**
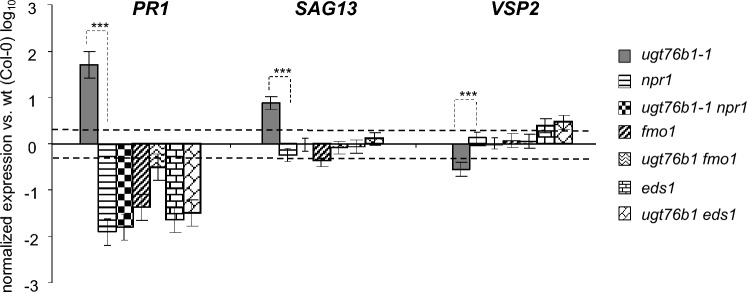
Marker gene expression in *ugt76b1* knockout after introgression of* npr1, fmo1*, and *eds1*. Gene expression of *PR1, SAG13,* and *VSP2* in four-week-old *ugt76b1-1* and *ugt76b1* double mutants with *npr1*, *fmo1, and eds1* was measured by RT-qPCR. Expression levels were normalized to *UBIQUITIN5* and *S16* transcripts; levels relative to Col wild-type plants are displayed. Arithmetic means and standard errors from log_10_-transformed data of three independent replicates from two separate experiments are displayed. The dashed, horizontal lines indicate a twofold change. Statistical analysis was performed by the software R using two-way analysis of variance (ANOVA; linear mixed effect models) followed by post hoc Tukey’s HSD test correction. ***P value ≤0.001

The loss of *UGT76B1* results in the antagonistic repression of the SA pathway and activation of the JA pathway (von Saint Paul et al. 2011). *ugt76b1 sid2-1*, *ugt76b1 npr1*, *ugt76b1 eds1*, and *ugt76b1 fmo1* double mutants were employed to test whether the impact of *ugt76b1* on the SA–JA crosstalk is influenced by *NPR1*, *EDS1*, and *FMO1*. The induction of SA marker *PR1* and suppression of JA marker *VSP2* of *ugt76b1* were relying on *SID2* (von Saint Paul et al. 2011). The enhanced expression of *PR1* and *SAG13* and the suppression of *VSP2*, a marker of *MYC2*/*JIN1*-mediated branch by *ugt76b1* are completely dependent on *NPR1* (Fig. 4). Similarly, the loss of *EDS1* and *FMO1* abolishes both the induction of *PR1* and *SAG13* and the suppression of VSP2 (Fig. 4), although there is a not significant tendency that *PR1* can be further induced in *fmo1* by introgressing *ugt76b1* (Fig. 4). Thus, the activation of the SA pathway and the suppression of the JA pathway by the loss of *UGT76B1* is dependent on SA and NHP biosynthesis and *NPR1* signaling.

### The early senescence upon loss of *UGT76B1* relies on *EDS1*, *FMO1*, and *NPR1*

**Fig. 5:**
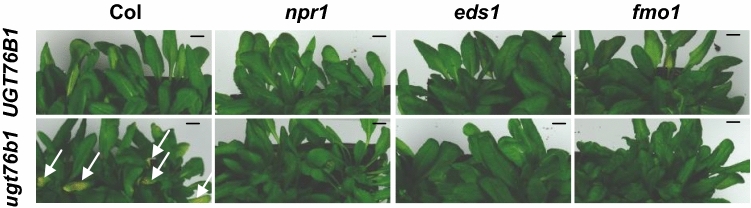
The impact of *UGT76B1* expression on the onset of senescence is dependent on *NPR1*, *FMO1,* and *EDS1*. Six-week-old wild type (Col), *ugt76b1*, *npr1*, *ugt76b1 npr1*, *fmo1*, *ugt76b1 fmo1*, *eds1*, and *ugt76b1 eds1*. Senescence is indicated by yellowing of leaves of *ugt76b1* (*arrows*), which is eliminated by the introgression of *npr1*, *fmo1*, or *eds1*. Similar results were observed in independent growth campaigns. *Bar* 1 cm

The early senescence of *ugt76b1* requires basal SA level (von Saint Paul et al. 2011). The master regulator *NPR1* was reported to positively influence senescence (Yoshimoto et al. 2009; Zheng and Dong 2013). *EDS1* regulates plant immunity via both *SID2*-mediated SA synthesis and an *SID2*-independent manner upstream of *FMO1* (Bartsch et al. 2006). *FMO1* controls SAR in both SA-dependent and independent manners (Bernsdorff et al. 2016; Hartmann and Zeier 2018; Hartmann et al. 2018). Therefore, to explore the dependence of the senescence phenotype of *ugt76b1* on *NPR1*, *EDS1*, and *FMO1* aging was observed for *ugt76b1 npr1*, *ugt76b1 eds1*, and *ugt76b1 fmo1* double mutants. The early senescence of *ugt76b1* is completely relying on *NPR1*, *EDS1*, and *FMO1* (Fig. 5).

## Discussion

**Fig. 6:**
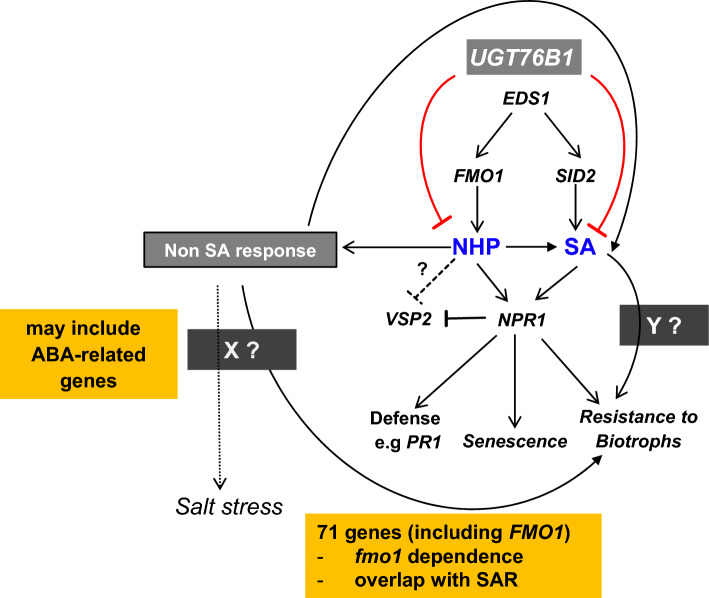
*UGT76B1*’s impact on defense pathways. NHP and SA are synthesized by FMO1 and SID2, respectively, and controlled by a common regulator EDS1. UGT76B1 glucosylates NHP and SA and thereby inhibits their immune-activating action. Thus, the loss of *UGT76B1* activates *SID2*- and *NPR1*-dependent SA signaling. Within the SA pathway, some genes are regulated dependent on *SID2*, however independent from *NPR1*, suggesting the existent of an additional path or factor (“Y”). Furthermore, transcriptome analysis of *ugt76b1* revealed another group of non-SA responsive genes appearing to be *SID2*- and *NPR1*-independent, this group also includes ABA-regulated genes. The non-SA responses may regulate ABA-related abiotic stresses for instance salt stress as well. Non-SA responsive, however *SID2*- and *NPR1*-independently induced genes such as *WRKY55* may regulate SA biosynthesis. Compared with Hartmann et al. (2018), 71 *ugt76b1*-upregulated genes overlapped with SAR-induced genes, which are completely dependent on *FMO1*. Furthermore, many genes within non-SA responsive group showed regulation at least partially relying on functional *NPR1*. The suppression of the JA marker *VSP2* in the *ugt76b1* mutant scenario requires *EDS1*, *FMO1*, and *NPR1* probably due to the repressive effect of SA pathway. Both *SID2*-dependent and independent defense responses and senescence development of *ugt76b1* rely on *FMO1*. The *dashed lines* indicate hypothetical relations

UGT76B1 competitively glucosylates and inactivates the immune-stimulating SA, ILA, and NHP and thereby keeps defense in check in naïve, uninfected plants. SA and NHP accumulate after infection and their abundance positively correlates with the resistance to pathogens. Thus, the enhanced SAR-like defense status of *ugt76b1* was primarily linked to a higher level of SA and NHP (Bauer et al. 2021; Cai et al. 2021; Holmes et al. 2021; Mohnike et al. 2021; von Saint Paul et al. 2011). To explore the dependence of the activated immunity of *ugt76b1* on SA or NHP and to discover potential SA-unrelated effects, genes altered by *ugt76b1* were first classified as SA-responsive or non-SA responsive according to the responsiveness to exogenous SA and the SA analogue BTH. Most of the SA-responsive genes of *ugt76b1* show SID2 and NPR1 dependence based on the responsiveness of *npr1* and *sid2* to pathogen infections (Supplementary Table 1). Thus, *UGT76B1* has a key role in suppressing a set of SA-responsive genes, which are mainly regulated via* SID2* and *NPR1*. The important role of *UGT76B1* in suppressing the SA-responsive group is consistent with the function of UGT76B1 to glucosylate SA. NPR1 independence within the SA signaling pathway may require the WHIRLY (WHY) transcription factor family (Desveaux et al. 2004, 2005; Vlot et al. 2009). However, very few genes of the SA-responsive group showed *SID2* dependence, yet *NPR1* independence in response to pathogen infections, thereby suggesting the existence of an independent link (Supplementary Table 1; Fig. 6: factor Y).

Moreover, many genes among the SA-responsive group can be regulated by pathogen infections independent from both *SID2* and *NPR1* (Supplementary Table 1 and 11), suggesting the existence of an independent signaling pathway that can target the same genes as SA. Since both SA and NHP accumulated to a higher level in *ugt76b1*, NHP may be the relevant signal. Indeed, 47 among 51 SA-responsive, but independently from *SID2* and *NPR1 ugt76b1*-upregulated genes and 39 out of 53 *ugt76b1*-suppressed genes overlapped with SAR-induced genes (Hartmann et al. 2018; Supplementary Table 11). All these SAR-regulated genes are controlled by *FMO1*, which is responsible for producing NHP, suggesting that NHP itself is involved in this signaling pathway. *FMO1* and its product NHP are known to regulate SA biosynthesis to enhance plant defense and SA is required for fully realizing the NHP-triggered defense (Vlot et al. 2021; Yildiz et al. 2021) (Fig. 6). However, NHP also can regulate defense responses independent from SA biosynthesis, i.e., it can still induce plant immunity in *sid2* (Bernsdorff et al. 2016; Yildiz et al. 2021; Zeier 2021). Moreover, NHP accumulation is independent from *SID2*, i.e., SA biosynthesis (Bauer et al. 2021; Hartmann and Zeier 2018; Hartmann et al. 2018; Zeier 2021). Thus, the enhanced level of NHP of *ugt76b1* plants may point to the existence of an SID2-independent defense regulation apart from the immediate effect of the missing NHP glucosylation by UGT76B1 (Bauer et al. 2021; Holmes et al. 2021; Mohnike et al. 2021). Consistently, many genes of the non-SA responsive group of *ugt76b1* are highly responsive to pathogen infections independent from *SID2* (Table 2 and Supplementary Table 2). SAR triggerd by exogenous application of NHP requires functional *NPR1* (Yildiz et al. 2021). Among the non-SA-responsive group regulated by *ugt76b1*, many genes indeed showed *NPR1*-dependent; however, *SID2-independent* upregulation responding to pathogen infections, suggesting that *ugt76b1*-triggered non-SA responsive plant defense is caused by NHP accumulation and at least partially relies on *NPR1* (Fig. 6). However, the extent of intercellular hyphae development and oospore formation was significantly reduced by NHP when infecting *npr1* plants with the *oomycete Hyaloperonospora arabidopsidis*, suggesting the residual *NPR1*-independent defense response induced by NHP (Yildiz et al. 2021*)*. Seventy non-SA responsive genes of *ugt76b1*-regulated genes showed both *SID2*- and *NPR1*-independent pathogen responses. *FMO1* ranks top among the *SID2*/*NPR1*-independent defense genes upregulated by *ugt76b1* (Table 2). However, *FMO1* induction by SAR also shows *SID2* independence. The *SID2*-independent defense regulation in SAR is completely relying on *FMO1* and its product NHP (Bernsdorff et al. 2016; Gruner et al. 2013; Hartmann and Zeier 2018; Hartmann et al. 2018). Consistently, the resistance of *ugt76b1* against *Pseudomonas syringae* is partially dependent on both *SID2* and *NPR1* (Fig. 3), however, completely relying on *FMO1* (Bauer et al. 2021). Seventy non-SA responsive genes showing *SID2*/*NPR1*-independent pathogen responses (Table 2) overlap with SAR-upregulated genes, which are completely dependent on *FMO1* (Supplementary Table 9) (Hartmann et al. 2018). Moreover, there is a close coexpression between *UGT76B1* and *FMO1* (Supplementary Fig. 2). Thus, except the influence caused by the lost ability to glucosylate SA in *ugt76b1*, the impact of *ugt76b1* on plant defense has also an *SID2*/*NPR1*-independent component regulated via* FMO1* (Fig. 6), since its product NHP can be competitively glucosylated by UGT76B1 as well. FMO1 and its product NHP could be responsible for the *SID2*/*NPR1*-independent pathogen resistance of *ugt76b1* (Fig. 6).

Besides *FMO1*, 70 further, *ugt76b1*-upregulated genes can be induced by pathogen infections independent from *SID2* and *NPR1* (Table 2). NHP is known to activate SA biosynthesis genes (Vlot et al. 2021; Yildiz et al. 2021).These genes (Fig. 6: X factor) may be the downstream targets of *FMO1* and NHP, which can further amplify the defense response for instance to regulate SA biosynthesis. For instance, *WKRY55* mediates defense response and senescence development through manipulating SA biosynthesis (Wang et al. 2020). *SRG1*, together with *SRG2* and *SRG3,* are positive regulators of SA-controlling plant immunity (Cui et al. 2021). Apart from manpulating SA signaling, some other X genes regulated by *ugt76b1* may impact defense by different mechanisms. For instance, *KTI1* inhibits cell death to result in the enhanced susceptibility towards pathogens (Li et al. 2008), whereas *CRK20* mediates the favorable apoplastic conditions to promote pathogen proliferation (Ederli et al. 2011). The camalexin biosynthesis-regulating gene *CYP71A12* favors the resistance by increasing the accumulation of camalexin (Lemarie et al. 2015), and overexpression of *RABA4C* causes resistance against pathogens by promoting the deposition of callose (Ellinger et al. 2014). The transcription factor *MYB15* encodes a positive regulator inducing lignin accumulation to fight against pathogens (Chezem et al. 2017). The gene *PUB22* ubiquitinates and degrades a positive regulator of PAMP-triggered immunity (Stegmann et al. 2012). The pathogen-induced CAM-binding protein-encoding gene *PICBP* is highly induced after pathogen infections (Reddy et al. 2003). The terpene synthase *TPS4* contributes to the resistance against pathogens by terpene production (Attaran et al. 2008). These examples suggest that the loss of *UGT76B1* triggers a broad activation of immunity including many aspects. Furthermore, two other ABA-related genes, *CSAP* and *JUL1*, were found to participate in ABA-mediated senescence and tolerance to drought stress, respectively (So et al. 2020; Yu et al. 2020). By now, their roles in immunity are not confirmed yet. This suggests that X factors may include ABA-mediated genes and X factors may regulate ABA-mediates responses such as salt stress (Fig. 6). Indeed, many ABA-responsive genes are regulated by SAR, however, still dependent on *FMO1* to be induced (Gruner et al. 2013). In agreement with this, the GO term enrichment also indicates that *ugt76b1*-upregulated genes are over-represented in genes related to “response to abscisic acid” and “response to salt stress” categories (Fig. 2a). Therefore, the enhanced immunity status of *ugt76b1* may be partially due to ABA-mediated responses as well and /or indicates an link of *UGT76B1* to abiotic stresses (Fig. 6), ABA-mediated abiotic stresses such as salt stresses require to be explored in *ugt76b1* mutants in future studies. Since the enhanced resistance of *ugt76b1* is fully determined by FMO1 (Bauer et al. 2021) and all these 70 genes are overlapping with *FMO1*-dependent SAR-induced genes, the *SID2*- and *NPR1*-independent regulation is likely to be controlled by *FMO1* and its product NHP. In the future, more signaling components downstream of *FMO1* should be explored as well.

Antagonism between SA and JA pathways is extensively studied and conserved in many different species (Pieterse et al. 2012). Treatment with SA or pathogen infection suppresses JA-regulated *VSP2* expression in *Arabidopsis* (Koornneef et al. 2008; Leon-Reyes et al. 2009), which also requires *NPR1* (Spoel et al. 2003). *SID2* is necessary for regulating SA–JA crosstalk, including *PR1* regulation, enhanced senescence, and suppression of *VSP2* by *ugt76b1* (von Saint Paul et al. 2011). Similar to *SID2*, *NPR1* is required for *ugt76b1* to suppress the JA pathway, e.g., *VSP2* expression (Figs. 4 and 6). Moreover, *EDS1*, upstream of both SA and NHP biosynthesis, and *FMO1*, known to regulate stress-induced SA biosynthesis upstream of *SID2* (Mishina and Zeier 2006; Vlot et al. 2021) are required for the induction of SA response and suppression of the JA response in *ugt76b1* as well (Figs. 4 and 5). Therefore, the need of *EDS1* and *FMO1* for *ugt76b1* to influence SA–JA crosstalk may be related to the impact of *ugt76b1* on SA biosynthesis. Furthermore, Yan et al. (2014) showed that MeJA treatment of *Arabidopsis* seedlings suppresses *ALD1* expression and Pip levels, suggesting a suppression of Pip biosynthesis by JA. In turn, Pip (or NHP) may confer direct suppression on JA pathway as well. Nevertheless, it cannot be excluded that *FMO1* and NHP may directly suppress the JA pathway of *ugt76b1*, independent from SA. The requirement of *NPR1*, *EDS1*, and *FMO1* in developing early senescence of *ugt76b1* may be due to the need of integrate SA pathway.

Together, *UGT76B1* impacts plant immunity by both *SID2*- and *NPR1*-dependent and independent regulation. The *SID2*- and *NPR1*-dependent regulation is mainly due to the lost ability of UGT76B1 to glucosylate SA, whereas the *SID2*- and *NPR1*-independent regulation is relying on *FMO1* and its product NHP. The identified *SID2*- and *NPR1*-independent defense genes among the non-SA-responsive group of *ugt76b1*-regulated genes illustrate the importance of an additional regulation not associated with SA signaling which is controlled by *UGT76B1 *via manipulation of NHP abundance.

### Supplementary Information

Below is the link to the electronic supplementary material.

Supplementary file1 (XLSX 330 KB)

Supplementary file2 (PPTX 130 KB)

## Data Availability

The raw data of the microarray expression are availabale at https://www.ebi.ac.uk/biostudies/arrayexpress/studies/ with ther code E-MTAB-13784.
