# Supplementary material for: SA and NHP glucosyltransferase UGT76B1 affects plant defense in both SID2- and NPR1-dependent and independent manner
Source: Plant Cell Rep. 2024 May 23;43(6):149. doi: 10.1007/s00299-024-03228-5 (PMC11116260; doi:10.1007/s00299-024-03228-5)
Supplement: Supplementary file 2 — Supplementary file2 (PPTX 130 KB) [file 299_2024_3228_MOESM2_ESM.pptx]

## Slide 1
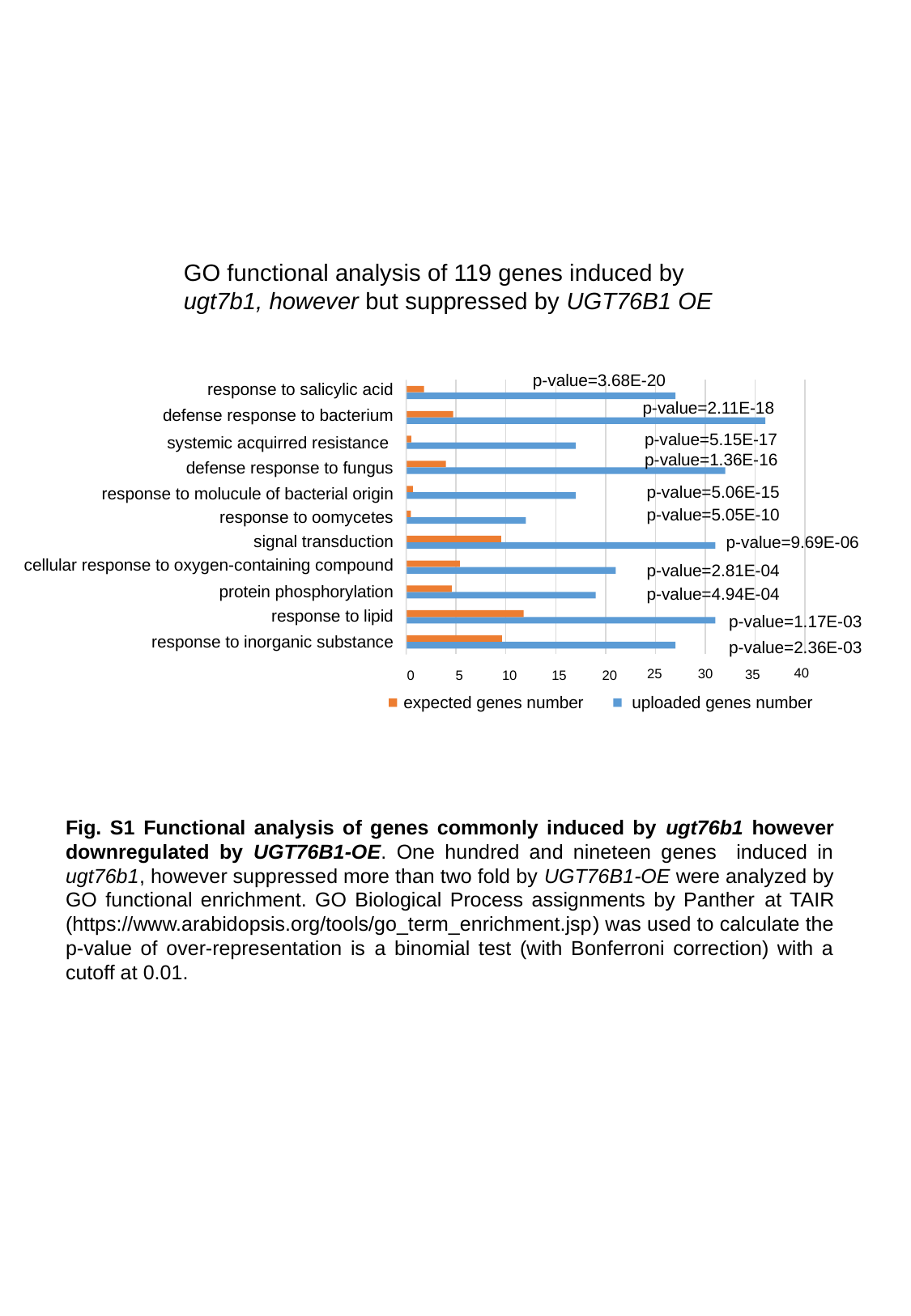

GO functional analysis of 119 genes induced by ugt7b1, however but suppressed by UGT76B1 OE
p-value=3.68E-20
response to salicylic acid
defense response to bacterium
systemic acquirred resistance
defense response to fungus
response to molucule of bacterial origin
response to oomycetes
signal transduction
cellular response to oxygen-containing compound
protein phosphorylation
response to lipid
response to inorganic substance
p-value=2.11E-18
p-value=5.15E-17
p-value=1.36E-16
p-value=5.06E-15
p-value=5.05E-10
p-value=9.69E-06
p-value=2.81E-04
p-value=4.94E-04
p-value=1.17E-03
p-value=2.36E-03
40
25
30
35
0
5
10
15
20
expected genes number
uploaded genes number
Fig. S1 Functional analysis of genes commonly induced by ugt76b1 however downregulated by UGT76B1-OE. One hundred and nineteen genes induced in ugt76b1, however suppressed more than two fold by UGT76B1-OE were analyzed by GO functional enrichment. GO Biological Process assignments by Panther at TAIR (https://www.arabidopsis.org/tools/go_term_enrichment.jsp) was used to calculate the p-value of over-representation is a binomial test (with Bonferroni correction) with a cutoff at 0.01.

## Slide 2
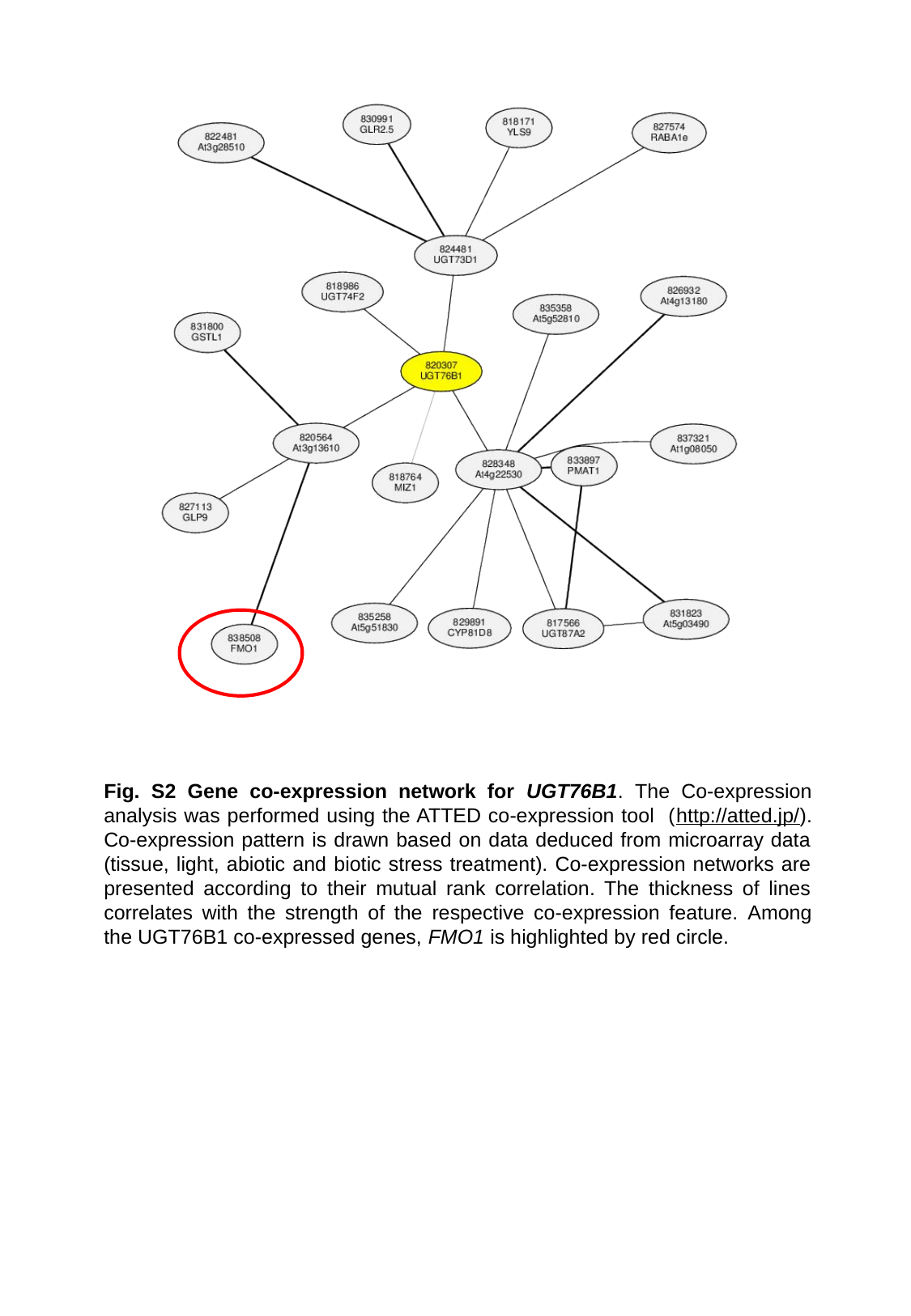

Fig. S2 Gene co-expression network for UGT76B1. The Co-expression analysis was performed using the ATTED co-expression tool (http://atted.jp/). Co-expression pattern is drawn based on data deduced from microarray data (tissue, light, abiotic and biotic stress treatment). Co-expression networks are presented according to their mutual rank correlation. The thickness of lines correlates with the strength of the respective co-expression feature. Among the UGT76B1 co-expressed genes, FMO1 is highlighted by red circle.
